# Impact of customary fluoride rinsing solutions on the pellicle’s protective properties and bioadhesion *in situ*

**DOI:** 10.1038/s41598-017-16677-8

**Published:** 2017-11-29

**Authors:** A. Kensche, J. Kirsch, S. Mintert, F. Enders, S. Pötschke, S. Basche, B. König, C. Hannig, M. Hannig

**Affiliations:** 1 Clinic of Operative and Pediatric Dentistry, Medical Faculty Carl Gustav Carus, TU Dresden, Fetscherstr. 74, D-01307 Dresden, Germany; 2 Clinic of Operative Dentistry, Periodontology and Preventive Dentistry, University Hospital, Saarland University, Building 73, D- 66421 Homburg/Saar, Germany

## Abstract

This study investigated the impact of customary fluoride based mouthrinses on the ultrastructure and the functional properties of the *in situ* pellicle, considering the prevention of erosion (8 volunteers) and initial biofilm formation (12 volunteers). Bovine enamel slabs were carried intraorally. After 1 min of pellicle formation, the subjects rinsed with elmex Kariesschutz (A), Dontodent Med Care (B), meridol (C) or elmex Zahnschmelzschutz Professional (D) for 1 min. *In situ* pellicle formation was continued up to 30 min/8 h before processing the slabs *in vitro*. Erosion was simulated by incubating the specimens in HCl (pH 3.0, 2.3, 2.0) for 120 s, measuring the kinetics of calcium/phosphate release photometrically; representative samples were evaluated by TEM and EDX. Bacterial adhesion was visualized fluorescence microscopically (DAPI/BacLight). Native enamel slabs or physiological pellicle samples served as controls. All investigated mouthrinses enhanced the erosion preventive pellicle effect in dependence of the pH-value. A significant decrease of Ca/P release at all pH values was achieved after rinsing with D; TEM/EDX confirmed ultrastructural pellicle modifications. All mouthrinses tendentially reduced bacterial adherence, however not significantly. The mouthrinse containing NaF/AmF/SnCl_2_ (D) offers an effective oral hygiene supplement to prevent caries and erosion.

## Introduction

On the basis of extensive clinical evidence it is indisputable, that regular fluoride application significantly decreases caries incidence as well as to some extent prevents the development of acid induced demineralization defects at the tooth surface^1–4^. However, the etiology, the morphology and pathogenesis of caries on the one hand and erosion derived dental hard tissue defects on the other differ considerably^5,6^. In this context, profound investigations of biomolecules’ adhesion as well as initial bacterial biofilm formation at the tooth surface under the influence of fluoride application *in situ* can provide valuable information about fluorides’ mechanism of action in preventive dentistry^7–9^. It must be assumed that all active agents applied to the oral cavity interact primarily with components of the omnipresent pellicle layer. The pellicle is formed by the selective adsorption of salivary proteins and glycoproteins to the tooth surface and it plays a key role protecting it against acid derived demineralization as well as providing bacterial receptors^10,11^. For this reason it is important to verify data gathered from various *in vitro* studies under *in situ* conditions^10,12^.

According to a widely accepted model conception, the application of fluoride containing preparations enforces the precipitation of a calcium-fluoride-like material at the tooth surface^3,13^. However, scanning electron microscopy has indicated, that the density and stability of such CaF_2_ precipitates is limited after the singular application of conventional monovalent fluorides *in vitro* and *in situ* ^3,7,14,15^. In case of severe erosive challenge, the suggested material deposits fail to serve as a sufficient physical barrier or mineral reservoir, respectively^3,15,16^. By contrast, concerning caries prevention, there is evidence for an antibacterial and antiadhesive effect of fluoride containing oral prophylactic agents. Certain fluoride components injuriously affect the metabolism of *streptococcus mutans*, inhibit bacterial enzymes’ activities and reduce bacterial adhesion to the tooth surface^7^.

The majority of customary mouthrinses contains inorganic sodium- or organic amine fluoride^17,18^. However, corresponding *in vitro* studies provide no unequivocal evidence for either sodium- or amine fluoride to have more significant protective effects at the tooth surface^15,17,19^. By comparison, the addition of stannous ions, either as SnF_2_ or SnCl_2_, present a promising advancement for erosion prevention, even under advanced erosive challenge^3,20–22^. Based on the results of numerous *in vitro* and few *in situ* studies, it is argued that stannous fluoride application alone or in combination with sodium- and amine fluoride generates a metal-rich layer at the tooth surface including Sn_2_(PO_4_)OH, Sn_3_F_3_PO_4_ and Ca(SnF_3_)^21,23–25^. While stannous fluoride is very susceptible to pH-dependent precipitation (pH > 4), provision of stannous ions in form of SnCl_2_ can increase the concentration of the functional additive to achieve greater effectiveness^26,27^. Despite the promising test results of *in vitro* studies, little has been published about the influence of *in situ* pellicle formation on the effects of fluoride- and stannous ions at the tooth surface and vice versa^7,10,11,21^. *In vitro*, mouthrinses with fluorides and particularly if supplemented by stannous ions appeared to modify the quantity and the composition of the pellicle proteins accumulated at the enamel surface^8,28^. This could enhance the pellicle’s resistance against acid-derived dissolution and could furthermore lead to a modified bacterial colonization^29^. However, so far, there is no published data about fluoride-associated effects on the *in situ* pellicle’s ultrastructure^30^.

Against this background, the present research work aimed to evaluate the impact of different customary fluoride containing mouthrinses on both the erosion protective- as well as the initial bacterial biofilm modulating properties of the *in situ* pellicle. As one part of the study, acid induced enamel demineralization was measured by an established *in situ*/*ex vivo* method. Separately, initial bacterial colonization *in situ* was evaluated fluorescence microscopically. Additionally morphological alterations of the pellicle were visualized by electron microscopy. Thus this study will make a contribution to the understanding of fluoride- and stannous ions associated effects at the tooth surface *in situ* under clinically relevant conditions.

## Methods

### Subjects and specimens

**Figure 1:**
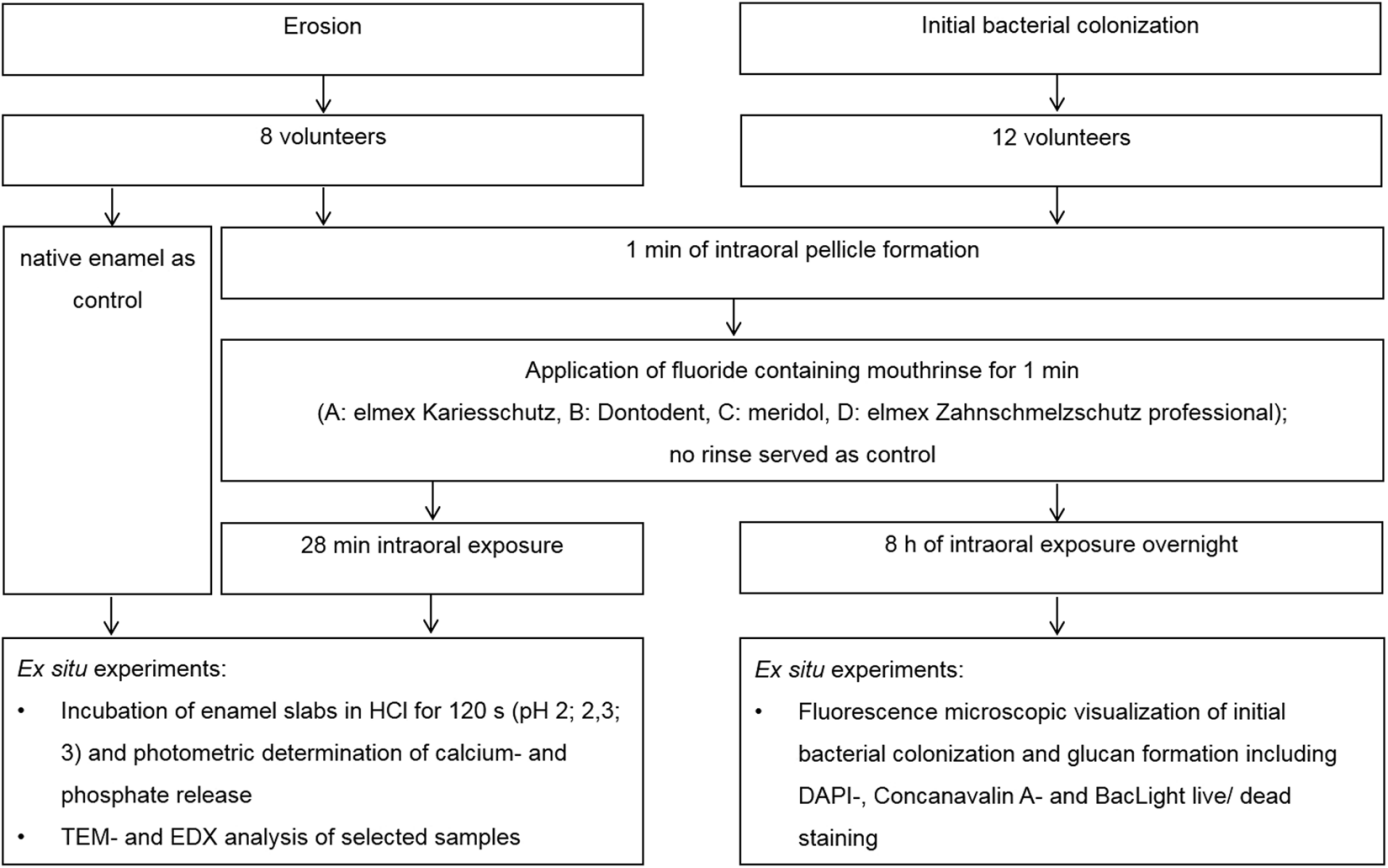
Flowchart of the experiments.

The present study includes two separate *in situ* investigations (Fig. 1). Eight volunteers agreed to participate in both, the experiments concerning the erosion preventive pellicle properties as well as the initial bacterial biofilm formation. Additional four volunteers only took part in the bacterial biofilm formation investigation (aged 25–43). All subjects had given their informed written consent about participation in the study. Any intraoral material exposure was approved by the ethics committee (vote: EK 147052013; Medical Faculty, Technische Universität Dresden, Germany) and all methods were carried out in accordance with the relevant guidelines. The volunteers were healthy non-smokers. At baseline, visual examination by an experienced dentist confirmed good oral health with no signs of caries, periodontal disease or unphysiological salivary flow. Individual upper jaw splints were adjusted for every participant, providing small cavities for specimens in the buccal region of 16, 15, 14, and 24, 25, 26. Bovine enamel slabs (5 mm diameter; 19,63 mm^2^ surface area, 1 mm thickness) were gained from incisor teeth of 2 year old cattle. The specimens were subjected to 37% phosphoric acid gel for 15 s on all surfaces except the outer enamel surface (Etching gel, DMG, Hamburg, Germany) and treated with Optibond FL (Kerr, Karlsruhe, Germany) which was light cured for 30 s. After wet-grinding and polishing the remaining enamel surface with up to 4000 grid abrasive paper, the resulting smear layer was removed by steam jet and ultrasonification with 3% NaOCl for 3 min. If the slabs showed no microscopically detectable signs of structural enamel alterations, they were washed twice for 5 min in destilled water activated by US, disinfected in 70% ethanol for 10 min (US), washed and stored in distilled water for 24 h. For intraoral exposure, the enamel slabs were placed in the provided cavities of the splints by polyvinyl siloxane impression material (Provil novo light regular set, Heraeus Kulzer, Germany), exposing only the specimens’ surface to the oral environment.

### Testsolutions

A variety of customary fluoride based mouthrinses were included in the study (Table 1). The selection comprised elmex Kariesschutz (GABA, Lörrach, Germany) containing 150 ppm sodium fluoride and 100 ppm amine fluoride (A), Dontodent Med Care Extra-Schutz (dm drogerie markt GmbH, Karlsruhe, Germany) with 500 ppm sodium fluoride (B), meridol (GABA GmbH, Lörrach, Germany) with a combination of 125 ppm amine fluoride and 125 ppm stannous fluoride (C) as well as elmex Zahnschmelzschutz professional (GABA GmbH, Lörrach, Germany) providing 275 ppm sodium fluoride, 125 ppm amine fluoride and 800 ppm stannous chloride (D). The pH-values of all mouthrinses were measured with a pH meter (Mettler-Toledo GmbH, Gießen, Germany) (Table 1).

**Table 1:** Principle composition of prophylactic agents used in this study.

Mouthrinse	Main ingredients	measured pH value
elmex Kariesschutz (GABA, Lörrach, Germany)	Water, 150 ppm Sodium fluoride, 100 ppm Olaflur (Amine fluoride) PEG-40 Hydrogenated castor oil, Aroma, Propylene glycol, Glycerin, Sodium benzoate, Levulinic acid, Sodium levulinate, Saccharin, Sodium anisate	4.4
Dontodent Med Care Extra-Schutz	Water, 500 ppm Sodium fluoride Glycerin, Sorbitol, Tetrasodium pyrophosphate, Cocamidopropyl betaine, Aroma, Sodium saccharin, Citric acid, Sodium benzoate, CI 47005, CI 17200	5.5
meridol	Water, 125 ppm Olaflur (Amine fluoride) 125 ppm Stannous fluoride PEG-40 Hydrogenated castor oil, Aroma, Xylitol, Sodium saccharin, Polyvinylpyrrolidon, CI 42051	4.2
elmex Zahnschmelzschutz Professional	Water, 375 ppm Sodium Fluoride 125 ppm Olaflur (Amine Fluoride) 800 ppm Stannous Chloride PEG-40 Hydrogenated Castor Oil, Glycerin, Sodium Gluconate, Aroma, Cocamidopropyl Betaine, Sodium Saccharin, Hydrochloric Acid	4.3

All experiments were carried out on separate days allowing a wash-out period of at least 48 hours in which the subjects performed their regular oral hygiene. Two hours before any test period, the participants were instructed to brush their teeth without toothpaste and to afterwards avoid any intake of food or drinks other than water.

### Determination of acid-induced calcium- and phosphate release

All *in situ* experiments to investigate the erosion preventive effects of the mouthrinses were scheduled between 7 and 12 a. m. In practice, the volunteers carried the splints for 1 min to allow *in situ* pellicle formation, subsequently rinsed with 8 ml of one of the selected mouthrinses for 1 min and finally carried the splints intraorally for another 28 min, ending up to a total exposure time of 30 min. Six enamel slabs were carried at a time. Unrinsed specimens with a 30 min *in situ* pellicle as well as native enamel specimens served as controls.

*In vitro*, the specimens were exposed to a 120 s erosive challenge by hydrochloric acid (pH 2, 2.3, 3), all tests were performed in duplicate. The acid-induced demineralization of the enamel specimens was determined by photometric analyses of dissociated calcium- and phosphate ions^31,32^. Therefore, the slabs were quickly removed from the splints, rinsed thoroughly with running water for 5 s and embedded with silicone impression material at the bottom of a 2 ml Eppendorff cup. 1000 µl of the acid solution were applied and circulated constantly by pumping with a 100 µl pipette (1 lift/s) to provide an even pH-value during incubation. Every 15 s 100 µl of the acid were removed for photometric analysis and replaced immediately by 100 µl of fresh acid. A double assay using the Arsenazo III- (Calcium) (Fluitest ®, Ca-A-II, analyticon, Lichtenfels, Germany) and the malachite green method (phosphate) was performed. The absorption was determined at λ = 650 nm according to standard curves. A suitable working solution was prepared of 100 mmol/l Imidazol buffer (pH 6.5) and 0.12 mmol/l Arsenazo III and for each measurement a volume of 10 µl from the sample was added to 100 µl Arsenazo-reagent. Furthermore, 0.045 mg of malachite green were dissolved in 100 ml aqua bidestilled water and mixed with 12.69 g ammonium molybdate dissolved in 300 ml HCl (4 mol/l). The solution was stirred for 30 min and filtered (pore size 0.22 µm). A volume of 10 µl from the sample was pipetted to 200 µl malachite-reagent and the absorption was measured after 15 min. All photometric measurements were performed as duplicate tests and the average absorption was calculated.

### Electron microscopic analysis

The ultrastructure of exemplary 30 min *in situ* pellicle samples with or without having been exposed to one of the mouthrinses and demineralization processes, respectively, were evaluated by transmission electron microscopy. Therefore, 2 corresponding 30 min pellicle samples of every treatment were generated just as for the calcium/phosphate-release measurement. After oral exposure and careful rinses of the enamel slabs with bidestilled water, they were either directly fixed in 1.5% formaldehyde −2.5% glutaraldehyde solution for 1 h at 4 °C or were incubated beforehand in HCl (pH 2.3) for 60 s, respectively. The fixed samples were washed 5 times in 0.1 M cacodylate buffer, then 1 ml of 1% osmium tetroxide was applied for 2 h to enable visualization of organic structures. After dehydration by immersion in an ascending ethanol-series the biofilm samples were embedded in Araldite CY212 (Agar Scientific Ltd, Stansted, United Kingdom), the existing enamel was decalcified by 0.1 M HCl and the samples were reembedded in Araldite. For TEM investigation, ultrathin sections of the pellicle samples were cut in series with an ultramicrotome (Ultracut E, Reichert, Bensheim, Germany). They were placed on pioloform coated copper grids (Plano, Wetzlar, Germany) and contrasted with uranyl acetate and lead citrate. Transmission electron microscopic analysis took place in TEM TECNAI 12 Biotwin (FEI, Eindhoven, The Netherlands) at magnification of up to 100,000 fold.

Additionally scanning electron microscopy coupled energy dispersive X-ray spectroscopy was performed in order to detect tin in the pellicle covered enamel surfaces. The investigated conditions included a 2 min *in situ* pellicle directly after the rinses, a 30 min *in situ* pellicle and a 30 min *in situ* pellicle which has been exposed to HCl of pH 2.3 for 1 min (n = 2 each). Samples without being rinsed served as control. After oral exposure the pellicle covered enamel specimens were gently rinsed with water and air – dried. After carbon sputtering the enamel surfaces were analyzed by a XL 30 ESEM (Philips, Eindhoven, The Netherlands) with an EDX device (Phoenix, EDAX INC., Mahwah, N.J., USA). at magnifications up to 5000 fold.

### Fluorescence Microscopy

The test protocol to investigate initial bacterial biofilm formation required the 12 participating volunteers to carry their splints for 8 hours overnight after 1 min of intraoral pellicle formation and rinses with 8 ml of one of the selected mouthrinses for 1 min. In this case four enamel slabs were exposed at a time to allow duplicate measurements. In the morning, the splints were rinsed carefully to remove any non-absorbed salivary remnants and the slabs were prepared for fluorescence microscopy. All fluorescence staining protocols were conducted as described previously^33,34^. The quantitative determination of both general bacterial adhesion and - viability were performed on the basis of 10 randomized microscopic ocular grid fields per sample^33^. Due to the defined size of the ocular grid fields (0.0156 mm²) calculation of the number of cells per square centimeter was allowed. All epifluorescent analyses were performed at 1000fold magnification (Axioskop II, ZEISS, Oberkochen, Germany).

### Bacterial adhesion and glucan formation

Adherent bacteria were visualized by DAPI (4′, 6-diamidino-2-phenylindole), which forms fluorescent units with adenine/thymidine-nucleic acids of double-stranded bacterial DNA. A combination of the dye with Alexa Fluor 574 conjugated Concanavalin A additionally visualized glucans as major extracelluar matrix molecules because the lectin binds specifically to the α-mannopyranosyl and α-glucopyranosyl residues of glucans^33^. The enamel slabs were washed in saline solution and were then covered with 0,5 ml of a solution which was prepared of 1,5 µl DAPI stock solution (1 mg/ml Methanol) and 10 µl ConvanavalinA stock solution in 498,5 µl of PBS. The slabs were incubated for 15 min in a dark chamber, were then rinsed with saline solution and left to air-dry, all at room temperature. Finally they were fixed to a slide and fluorescence microscopic investigation could be performed.

### BacLight viability assay

Additionally, LIVE/DEAD BacLight bacterial viability assay was performed. On the basis of two nucleid acid stains, the green fluorescent SYTO 9 stain (component A) and the red-fluorescent propidium iodide stain (component B) (Invitrogen, Molecular probes, Darmstadt, Germany), viable and dead bacteria could be differentiated. The stock solution consisted of equal amounts of component A (Syto9 1.67 mM/propidium iodide, 1.67 mM, 300 μL DMSO) and component B (Syto 9 dye, 1.67 mM/propidium iodide, 18.3 mM, 300 μL DMSO); to prepare a working solution, 2 µl were pipetted to 1 ml of saline solution. After the slabs were incubated in this solution for 10 min at room temperature it was rinsed off with saline solution and the slabs could be investigated under the fluorescence microscope using a fluorescein diacetate - and an ethidium bromide filter.

### Statistics

All data were statistically processed by SPSS 22.0 (IBM, Ehningen, Germany). The analysis of the erosion associated Ca/P release based on the mean value calculated from 16 enamel slabs of the 8 subjects in each subgroup after 120 s of incubation in HCl. Due to the lack of normal distribution, the Kruskal-Wallis test as well as additional pair-wise comparison by the Mann-Whitney U test were performed; after Bonferroni-Holm-correction the level of significance was set to p < 0.01. For the fluorescence microscopic data the mean value was calculated from 24 slabs of the 12 volunteers. Likewise, the Kruskal-Wallis- and Mann-Whitney U test were performed with p < 0.01 after Bonferroni-Holm-correction.

## Results

### Dissociation of calcium and phosphate

**Figure 2:**
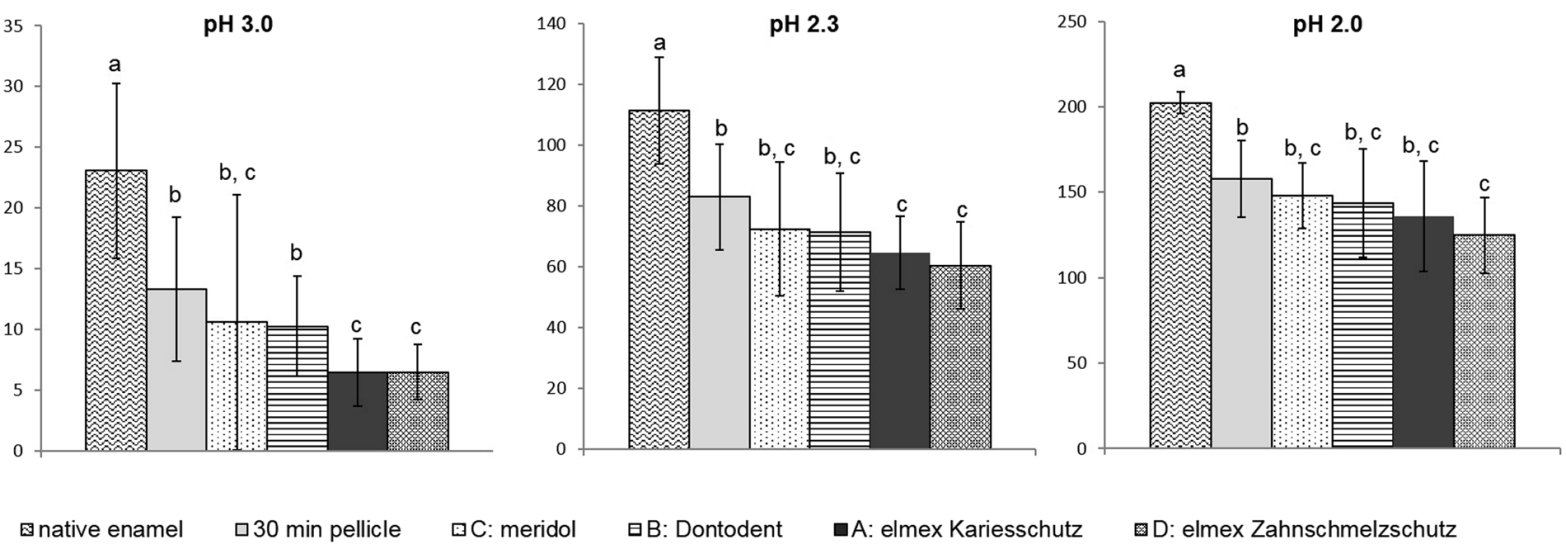
Cumulative calcium release of enamel slabs after 30 min of pellicle formation with and without application of different fluoride containing mouthrinses *in situ* and incubation in HCl (pH 3.0, 2.3, 2.0) for 120 s. Specimens without pellicle served as controls; n = 16 samples per subgroup. Data significantly different from each other are marked with different letters. At all pH-values, pellicle formation reduced calcium release significantly, which was enhanced by fluoride application. A significant increase (p < 0.01) of erosion-protective pellicle properties at all pH values was observed after application of the NaF/AmF/SnCl_2_-containing mouthrinse (Kruskal-Wallis test and Mann-Whitney-U-test).

**Figure 3:**
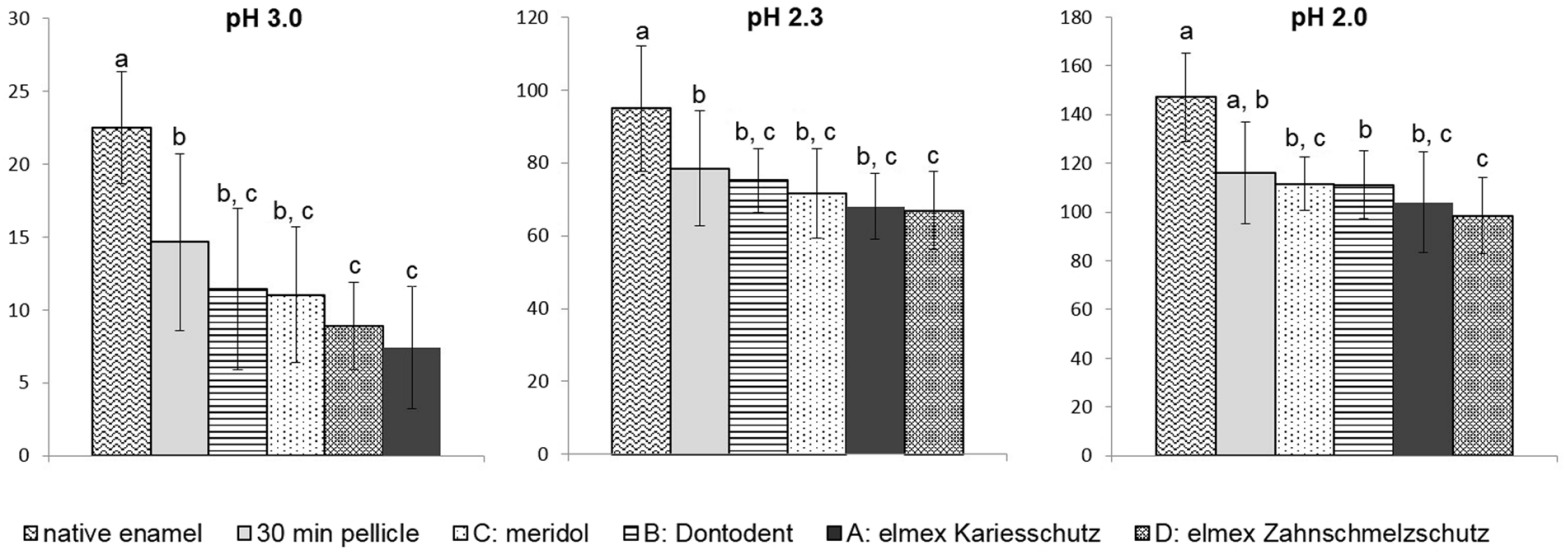
Cumulative phosphate release of enamel slabs after 30 min of pellicle formation with and without application of different fluoride containing mouthrinses *in situ* and incubation in HCl (pH 3.0, 2.3, 2.0) for 120 s. Specimens without pellicle served as controls; n = 16 samples per subgroup. Data significantly different from each other are marked with different letters. All fluoride based mouthrinses reduced the acid-induced phosphate release compared to the physiological 30 min pellicle, significance of this effect with p < 0.01 was confirmed for the NaF/AmF/SnCl_2_ containing mouthrinse (D) (Kruskal-Wallis test and Mann-Whitney-U-test).

As a common feature observed in all subgroups, the acid-induced dissolution of calcium and phosphate increased linearly to the acid exposure time as well as in dependence of the pH- value (data not shown). The cumulative calcium- and phosphate release after 120 s of incubation in HCl (pH 2, 2.3, 3) and their statistical evaluation are depicted in Figs 2 and 3. In comparison to native enamel specimens mineral loss was significantly reduced by the formation of a 30 min pellicle *in situ*. The mean concentration of dissociated calcium decreased by 37% at pH 3.0, 26% at pH 2.3 and 18% at pH 2.0. Similarly phosphate release was reduced by 33% at pH 3, 17% at pH 2.3 and 20% at pH 2. This demineralization-preventive effect of the *in situ* pellicle was unexceptionally enhanced by all investigated mouthrinses. However, in terms of significance, pairwise comparison of the mean mineral loss after 120 s of incubation in HCl revealed notable differences between the mouthrinses (Figs 2 and 3). Furthermore, it was shown that the pellicle modifying effect of the investigated preparations depended on the acid’s pH-value. At pH 3 and pH 2.3 the dissociation of calcium could significantly be reduced by the application of elmex Kariesschutz (A) (pH 3: −52%, pH 2.3: −22%; p < 0.01) and elmex Zahnschmelzschutz Professional (D) (pH 3: −51%, pH 2.3: −27.73%). However at pH 2, significance of this protective effect could only be confirmed for the mouthrinse containing the combination of NaF, AmF and SnCl_2_ (−22%, p < 0.01). The 500 ppm NaF-containing product (B) and the mouthrinse containing 125 ppm AmF and 125 ppm SnF_2_ (C) had no significant impact on the protective properties of the physiological pellicle at any of the investigated pH-values (p > 0.01). Pairwise comparison between the mouthrinses revealed significant differences among the mouthrinses only at pH 3 (p < 0.01). Both the NaF/AmF (A) - and the NaF/AmF/SnCl_2_ (D) – containing product were shown to promote the protective properties of the pellicle more effectively than the 500 ppm NaF-based mouthrinse (B).

In case of phosphate release similar tendencies were shown, although the pellicle-modifying effect was generally less pronounced. At pH 3, elmex Kariesschutz (A) as well as elmex Zahnschmelzschutz Professional (D) decreased phosphate release significantly compared to the physiological 30 min pellicle. At lower pH level, a significant decrease of phosphate dissociation compared to the unmodified 30 min pellicle could only be achieved by the application of the NaF/AmF/SnCl_2_ – containing product (D) (pH 2.3: −15%; pH 2: −15%; p < 0.01). According to the pairwise comparison, the NaF/AmF- containing product (A) was superior to the purely 500 ppm NaF - containing mouthrinse (B) at pH 3 and the combination of NaF/AmF/SnCl_2_ at pH 3 and pH 2.

### Ultrastructure and electron microscopic analysis

**Figure 4:**
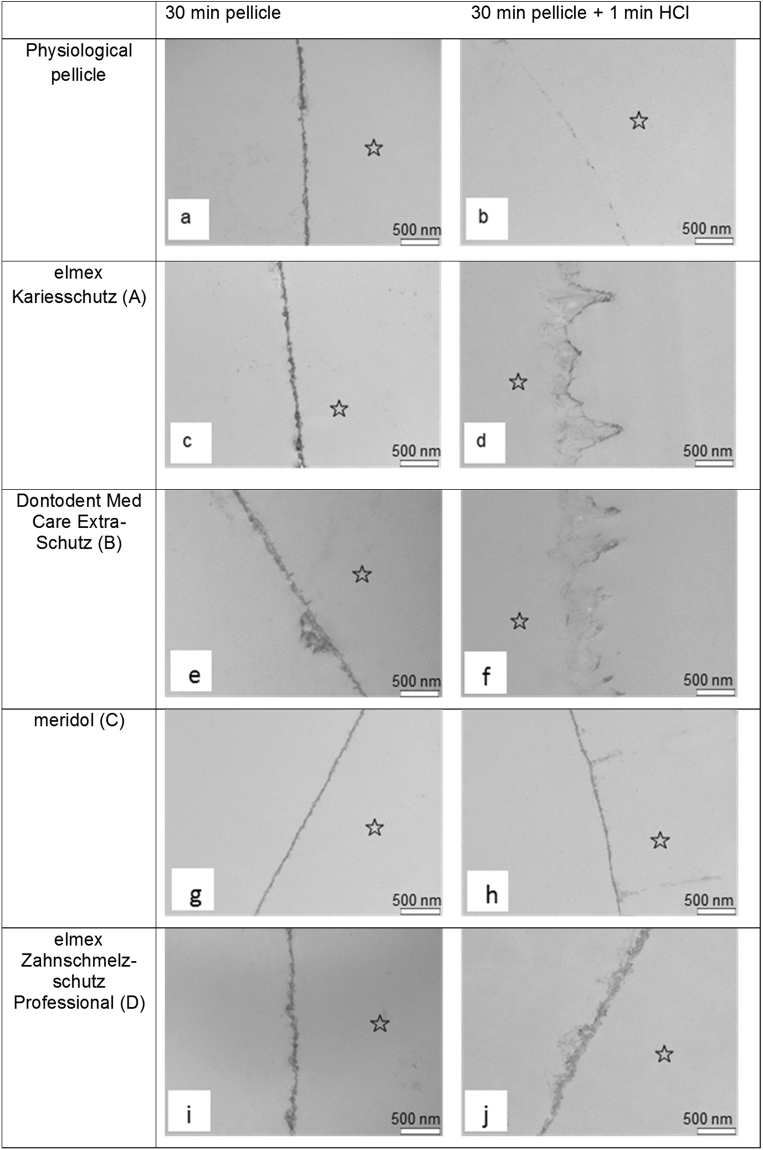
Representative TEM – images of 30 min *in situ* pellicle samples without (**a**,**b**) and with (**c**–**j**) pretreatment by one of the fluoridated mouthrinses before (**a**,**c**,**e**,**g**,**i**) and after incubation in HCl (pH 2.3) for 1 min (**b**,**d**,**f**,**h**,**j**). The physiologically formed 30 min *in situ* pellicle is characterized by a continuous electron dense basal layer as well as a thin granular second layer (**a**). Basically, neither of the applied mouthrinses appeared to notably alter the pellicle’s ultrastructure compared to the control (**c**,**e**,**g**,**i**). However, rinsing with elmex Zahnschmelzschutz Professional and to some extend also with meridol apparently strengthened the pellicle against acid-derived dissolution by HCl (pH 2.3) (**h**,**j**). In case of elmex Zahnschmelzschutz Professional, the investigated pellicle sample was, although less electron dense, approximately 80 nm thick and showed no signs of disrupture. In contrast, rinses with elmex Kariesschutz or Dontodent did not remarkably improve the pellicles’ resistance against erosive destruction and stria-like demineralisation-associated infiltrations of the enamel were visible. Please note that the former enamel site is marked with an asterisk as it was removed during the preparation process. Original magnification: 30,000 fold.

Transmission electron microscopic investigations were performed on 30 min *in situ* pellicle samples with or without having been exposed to one of the fluoridated mouthrinses in order to identify potential ultrastructural modifications (Fig. 4). In all cases, pellicle formation has occurred and a continuous up to 40 nm thick proteinaceous layer could be detected on all of the investigated enamel slabs. The physiological accumulation of an electron-dense basal layer and a more inhomogenous second granular layer has once again been confirmed and none of the mouthrinses appeared to notably affect this characteristic ultrastructural appearance. However, after rinses with elmex Zahnschmelzschutz Professional parts of the pellicle appeared to be less condensed (Fig. 4i). More distinct ultrastructural differences were detected after selected 30 min pellicle samples were incubated in HCl pH 2.3 for 1 min. As observed previously, the performed acidic challenge led to an almost complete destruction of the physiological 30 min pellicle. Pellicle modification neither with elmex Kariesschutz (A) nor with the 500 ppm NaF containing mouthrinse (B) increased the pellicle’s susceptibility against acid-derived dissolution. Indeed, the incubation in HCl pH 2.3 for 1 min notably affected the superficial enamel surface causing striae-like lesions of up to 800 nm depth (Fig. 4d,f). It is remarkable, that if rinses with the fluoridated mouthrinses were performed, organic material appeared to line these striae immediately. By comparison, if the meridol-modified 30 min *in situ* pellicle was exposed to the acid, large parts of a thin basal pellicle layer remained continuous (Fig. 4h). However, similar striae-like demineralisation lesions as described above were identified. Most distinct ultrastructural differences when compared to the physiological control were visible after rinsing with elmex Zahnschmelzschutz Professional. Even after being exposed to the acid for 1 min, an approximately 80 nm thick pellicle layer could still be detected. Compared to the unetched sample the pellicle appeared less electron dense and rather loose, but continuous and with no signs of enamel infiltration (Fig. 4j).

Additional energy-dispersive X-Ray spectroscopy confirmed an accumulation of tin at the tooth surface after rinsing with the 125 ppm SnF_2_- as well as the NaF/AmF/SnCl_2_ containing mouthrinse. Immediately after the rinse with meridol (C), the measured tin content in the sample amounted to 2.07 ± 0.61 wt% while rinsing with the NaF/AmF/SnCl_2_ containing preparation (D) generated a tin content of 1.62 ± 0.09 wt%. Further intraoral exposure up to 30 min changed the tin content to 1.16 ± 0.02 wt% for the 125 ppm SnF_2_ treated samples and 1.38 ± 0.15 wt% for the samples having been rinsed with 800 ppm SnCl_2_. After incubation of the 30 min pellicle samples in HCl pH 2.3 for 1 min, the tin content amounted to 1.39 ± 0.06 wt% for the samples that have been rinsed with meridol (C) and 1.25 ± 0.25 wt% for the samples that have been exposed to the NaF/AmF/SnCl_2_ containing mouthrinse. By contrast, no tin was detected in any of the samples that had been exposed to elmex Kariesschutz (A), the NaF-containing mouthrinse (B) or no rinse at all.

### Initial Bacterial colonization

**Figure 5:**
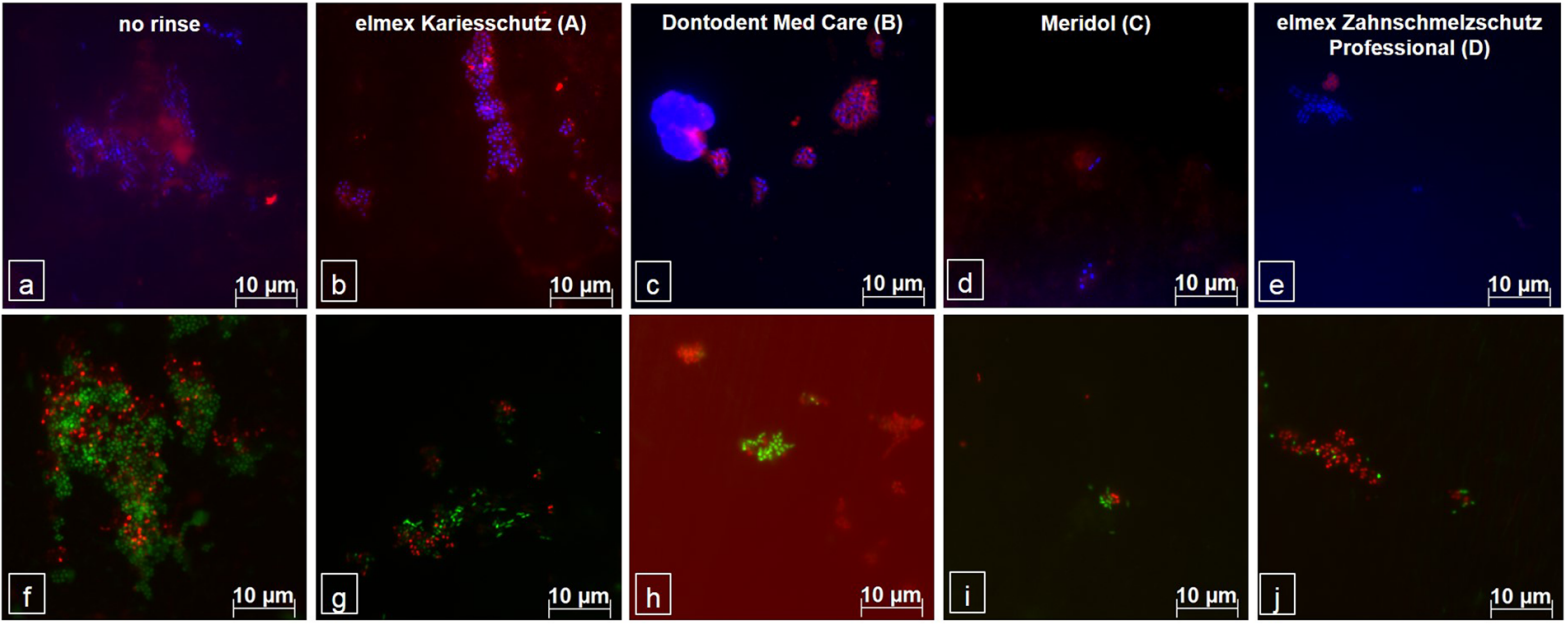
Representative images of fluorescence microscopic visualization of bacteria by DAPI- (**a**–**e**/blue) and BacLight- (**f**–**j**) staining, as well as glucan visualization by Concanavalin A (**a**–**e**/red). After 8 hours of intraoral exposure, dense bacteria – glucan agglomerates were detected on the unrinsed enamel slabs (**a**). Live/Dead staining confirmed a high proportion of vital bacteria (**f** - green). In contrast, bacterial adhesion as well as corresponding glucan formation was notably reduced by all fluoride-containing mouthrinses (**b**–**e**), a clear shift of the distribution of vital and dead bacteria could not be shown (**g**–**j**). After rinses with meridol (C) or elmex Zahnschmelzschutz Professional (D) only isolated bacterial accumulations were detected (**d**,**e**,**i**,**j**).

**Figure 6:**
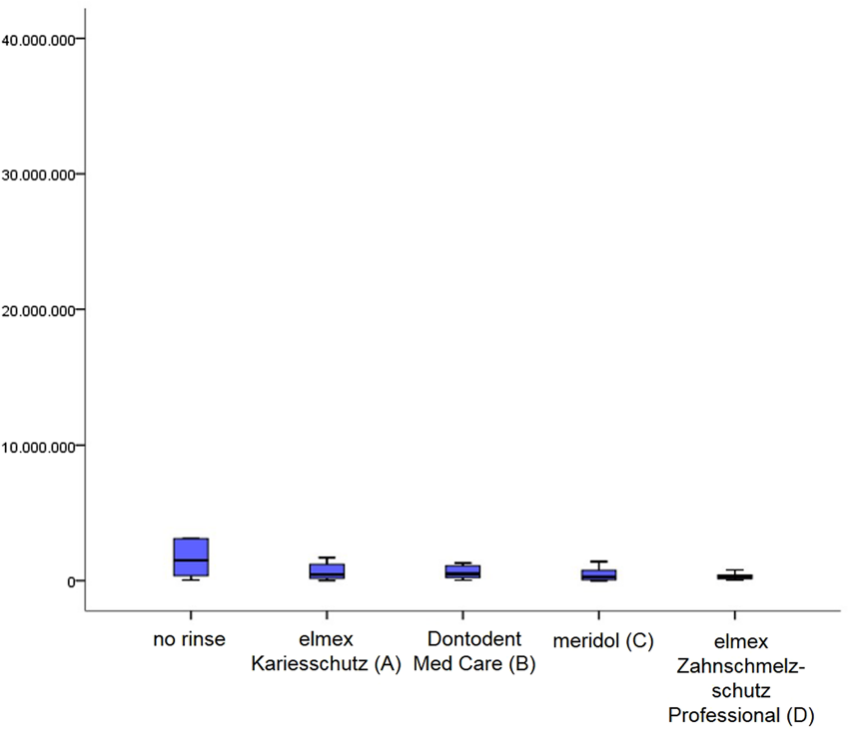
Influence of different fluoride containing mouthrinses on initial bacterial adhesion to enamel *in situ*, visualized by DAPI – staining. After 1 min of pellicle formation participants rinsed with 8 ml of one of the investigated mouthrinses for 1 min and continued carrying the splints with the specimens for 8 hours overnight. Samples exposed intraorally without rinsing served as control. All labeling was carried out in duplicate for n = 12 subjects. Although no significant difference was confirmed by the Kruskal-Wallis test (p < 0.01) a clear tendency to hamper initial bacterial adhesion was revealed for all investigated mouthrinses, the AmF/SnF_2_ (C)- and NaF/AmF/SnCl_2_ (D)– containing mouthrinse showing the strongest effects. Additional pairwise comparison by the Mann–Whitney U test yielded no significant difference between the rinses.

Visualization of general bacterial adhesion after 8 hours of intraoral biofilm formation as well as differentiation of the bacteria viability were achieved (Fig. 5). A significant reduction of the total amount of detectable bacteria could not be proven for any of the preparations (p ≤ 0.01) (Fig. 6). However, DAPI-staining revealed a clear tendency that in contrast to the control, all fluoride containing mouthrinses hampered an extensive bacterial colonization of the enamel slabs. Especially after rinses with elmex Zahnschmelzschutz Professional (D) or meridol (C), adhering bacteria appeared to be less densely aggregated at the tooth surface and glucans could hardly be detected (Fig. 5). Closer differentiation between viable and dead bacteria by BacLight Live/Dead staining reinforces the impression of a general bacterial colonization reducing effect of all fluoride containing rinses compared to the control (p ≤ 0.01). However, the proportion of viable and dead bacteria appeared not to be affected by any of the investigated mouthrinses.

## Discussion

In fact, although literature is replete with publications about the positive impact of fluorides on caries prevention and dental health^9,35,36^, the present study is one of few analyzing fluoride- and tin associated bioadhesion processes at the tooth surface under *in situ* conditions^20,21^. It is a unique feature of this study that both, the initial bacterial colonization and the erosion preventive capacity of the *in situ* pellicle were investigated after performing mouthrinses with different fluoride containing preparations. In this context, special focus was laid on the ultrastructural modification of the pellicle. On the basis of the present data, general oral hygiene recommendations can be adapted more precisely, especially since priority was given to the broad clinical relevance of the investigated mouthrinses. A variety of popular customary over the counter mouthrinses differing in fluoride- and functional compounds, price and addressed target group were deliberately chosen as test solutions in this study. Different concentrations as well as combinations of anorganic NaF and SnF_2_, organic AmF and the additive SnCl_2_ were contained in the selected products.

Despite the sparse comparability of preliminary studies considering the efficacy of fluorides to prevent erosive mineral loss at the tooth surface, there is evidence that the addition of stannous ions remarkably increases the rinsing solutions’ protective potential^3,21,37–39^. The present study succeeded to substantiate these indicative findings under *in situ* conditions. According to the acid-induced Ca/P-dissociation measurements it can be concluded that all fluorides tendentially obtain or enhance the protective pellicle properties in dependency of the pH-value^10^. However, the limited significance as well as the clear decrease of these effects under severe erosive conditions show that a potential accumulation of KOH-soluble calcium fluoride at the tooth surface or pellicle modifications, respectively, must be limited after singular application of NaF-, AmF- or SnF_2_ at concentration of 125–500 ppm^28,30^. Proteomic pellicle investigations *in vitro* suggested, that fluoride application promotes the adsorption of specific proteins such as mucins, histatin 3 and PRP at the enamel surface, influencing the pellicle’s density as well as molecular interactions^28^. Since these bioadhesion processes are to a major extend based on electrostatic interactions, differences might occur due to the fluoride-bound cations. As confirmed in this study, 500 ppm of sodium fluoride alone (B) could only slightly modify the erosion-preventive effect of the physiological pellicle. In comparison, the amphiphilic properties of amine fluoride such as contained in elmex Kariesschutz might have increased its affinity to pellicle proteins resulting in a slower clearance and a modification of the pellicle composition or –density, respectively^37,40^. Certainly, only the NaF/AmF/SnCl_2_-containing mouthrinse (D) could reduce the acid-induced calcium- and phosphate release of the physiological *in situ* pellicle significantly even under severe erosive conditions. This effect was more pronounced for phosphate release than for calcium release which could be due to a higher retention of calcium in a fluoride modified pellicle^28^. The obvious superiority of the product containing 800 ppm SnCl_2_ to prevent acid-induced mineral loss at pH 2.0 might, in theory, be attributed to the deposition of stannous ions directly at the tooth surface^16^. However, it is well known, that the immediately formed proteinaceous *in situ* pellicle not only masks enamel surface properties but must have also been penetrated by the stannous ions^10,41^.

In this context the performed TEM-investigation and the EDX-analysis provide some notable evidence. To the best knowledge of the authors, a systematic analysis of ultrastructural modifications of the *in situ* pellicle by different fluoride components has so far never been performed. By contrast, the oralprophylactic properties of several functional agents have already been correlated with an alteration of the pellicle’s ultrastructure^29–31^. In general, none of the applied fluoride containing mouthrinses without stannous ions appeared to notably affect the thickness or electron density of the *in situ* formed 30 min pellicle, thus a considerable impact of fluorides on the pellicle’s ultrastructure can be questioned. By comparison, after rinsing with the additionally 800 ppm SnCl_2_ containing mouthrinse, parts of the *in situ* formed pellicle appeared slightly less condensed which was possibly due to an accumulation of tin compounds. More indicative findings were revealed from the samples that have been exposed to HCl pH 2.3. As expected, acid exposure led to an obvious dissolution of the physiological pellicle which matches the results of the Ca/P measurements. Neither rinsing with a NaF- nor with a NaF/AmF containing mouthrinse did notably strengthen the pellicle’s resistance against acid derived damage. However, the amount of dissociated calcium and phosphate was still reduced if compared to the control (Figs 2 and 3). In this context it has to be pointed out that the corresponding TEM-images revealed an adhesion of proteins at the surface of the demineralization defects giving them a striae-like appearance (Fig. 4d,f). Considering, that TEM only visualizes proteinaceous components, it can be concluded that the presence of fluoride ions or their potential affinity to the enamel surface might have enhanced the immediate reattachment of protein-components at the tooth surface *in situ* ^8^. With regard to the pellicle’s protective properties, this can be rated as a valuable reparation process which substantiates the superiority of any fluoride containing mouthrinse towards their omission^10^. Nevertheless, most significant erosion preventive effects must be attributed to the amount of stannous ions contained in the fluoride based mouthrinse. In contrast to the control or any of the other mouthrinses, incubation of the NaF/AmF/SnCl_2_ – modified pellicle samples in HCl did not result in a notable damage or loss of the pellicle’s ultrastructure and there were no signs of a demineralized enamel surface. These observations correspond with the determined Ca/P release, which was the only one to be significantly reduced at all pH-values when compared to the unrinsed physiological pellicle.

According to the performed EDX-analysis, rinsing with the NaF/AmF/SnCl_2_ – containing mouthrinse caused a demonstrable accumulation of tin at the tooth surface which was more or less the same after 30 min of pellicle formation *in situ* and which only slightly decreased after 1 min of acid exposure. By comparison, rinsing with the 125 ppm SnF_2_ containing mouthrinse initially caused an obvious accumulation of tin in the investigated samples which appeared to decrease considerably during 30 min of *in situ* pellicle formation. It is to be assumed that the notable differences between the two Sn-containing mouthrinses (C, D) considering their erosion-preventive effect are due to the different concentrations of stannous ions. The availability of stannous ions at the tooth surface appears to have a strong impact on a tin- rather than fluoride derived erosion-preventive effect of the NaF/AmF/SnCl_2_-containing mouthrinse. Particularly under strong erosive conditions, the protective effect of purely fluoride-based mouthrinses appears to be limited. For patients showing progressing signs of erosive tooth demineralization the use of the NaF/AmF/SnCl_2_- containing mouthrinse (D) should be recommended to inhibit further acid-induced demineralization. The indisputable relevance of this topic is substantiated by a previous publication by the American Dental Association which shows the strikingly wide distribution of extremely erosive beverages (pH < 3)^42^.

Appropriate daily oral health care preparations ideally provide protective effects against both erosive as well as bacterially derived dental hard tissue defects. Accordingly, it could be confirmed that all tested fluoride based mouthrinses showed a strong tendency to hamper bacterial adhesion to the pellicle covered enamel surface. In detail, uncertainty remains about the exact impact of fluorides on the pellicle components and functions *in situ* ^7^. Previous investigations have clearly demonstrated an antibacterial effect of the NaF/AmF based mouthrinse (B) on *streptococcus mutans* suspensions’ viability^7,43^. However, it is conceivable, that the pellicle’s electrostatic interactions with salivary components and bacterial cells are altered by fluorides^7,44^. In this context, the different fluoride cations and their individual affinity to the pellicle components must be considered. Especially amine fluoride has been suggested to demonstrably change the pellicle’s hydrophobicity and therefore influence initial bacterial adhesion processes^44^. In this study, the AmF/SnF_2_ (C)- and NaF/AmF/SnCl_2_ (D) containing mouthrinses appeared to reduce initial bacterial adhesion most effectively. With the information of the electronmicroscopic images in mind, this might also be attributed to an alteration of the pellicles physicochemical properties (Figs 4–6). However, no clearly noticeable difference regarding their influence on initial bacterial adhesion could be revealed between the different mouthrinses and their contained fluoride components.

In summary, our data doubtlessly reinforce the suitability of fluoride based mouthrinses as a supplement of daily oral hygiene measures. Within the wide range of commercially available preparations, the mouthrinse containing the combination of NaF, AmF and SnCl_2_ appeared to be the most effective one to prevent erosion at pH 2. In particular, it should be recommended in case of severe erosive challenge or if patients already show erosion derived dental hard tissue defects. All mouthrinses tendentially reduced bacterial adhesion. Merging the results of this study it can be suggested that the formation and therewith physicochemical properties of the *in situ* pellicle are altered by fluorides and that the additional application of higher concentrations of stannous ions application increases the protective pellicle properties. Distinct ultrastructural pellicle modifications were only revealed for AmF/SnF_2_ or NaF/AmF/SnCl_2_ treated *in situ* pellicles after acid exposure. Future studies should on the one hand investigate the pure substances’ effects at the tooth surface. On the other hand, the *in situ* effects of iterative fluoride application protocols also have to be taken into consideration.

## References

[1] Tenuta LM, Cury JA (2010). Fluoride: its role in dentistry. Brazilian oral research.

[2] Brambilla E (2001). Fluoride - is it capable of fighting old and new dental diseases? An overview of existing fluoride compounds and their clinical applications. Caries Res.

[3] Lussi A, Carvalho TS (2015). The future of fluorides and other protective agents in erosion prevention. Caries Res.

[4] Marinho VC (2009). Cochrane reviews of randomized trials of fluoride therapies for preventing dental caries. European archives of paediatric dentistry: official journal of the European Academy of Paediatric Dentistry.

[5] Lussi A, Carvalho TS (2014). Erosive tooth wear: a multifactorial condition of growing concern and increasing knowledge. Monographs in oral science.

[6] Bowen WH (2016). Dental caries - not just holes in teeth! Aperspective. Molecular oral microbiology.

[7] Hannig C (2013). Effect of conventional mouthrinses on initial bioadhesion to enamel and dentin *in situ*. Caries Res.

[8] Siqueira WL, Bakkal M, Xiao Y, Sutton JN, Mendes FM (2012). Quantitative proteomic analysis of the effect of fluoride on the acquired enamel pellicle. PloS one.

[9] Lussi A, Hellwig E, Klimek J (2012). Fluorides - mode of action and recommendations for use. Schweiz Monatsschr Zahnmed.

[10] Hannig M, Hannig C (2014). The pellicle and erosion. Monographs in oral science.

[11] Uhlen MM, Mulic A, Holme B, Tveit AB, Stenhagen KR (2016). The Susceptibility to Dental Erosion Differs among Individuals. Caries Res.

[12] Hannig M, Joiner A (2006). The structure, function and properties of the acquired pellicle. Monographs in oral science.

[13] Larsen MJ, Richards A (2001). The influence of saliva on the formation of calcium fluoride-like material on human dental enamel. Caries Res.

[14] Koeser J, Carvalho TS, Pieles U, Lussi A (2014). Preparation and optimization of calcium fluoride particles for dental applications. Journal of materials science. Materials in medicine.

[15] O’Toole S, Bartlett DW, Moazzez R (2016). Efficacy of sodium and stannous fluoride mouthrinses when used before single and multiple erosive challenges. Australian dental journal.

[16] Magalhaes AC, Wiegand A, Rios D, Buzalaf MA, Lussi A (2011). Fluoride in dental erosion. Monographs in oral science.

[17] Ganss C, Schlueter N, Hardt M, Schattenberg P, Klimek J (2008). Effect of fluoride compounds on enamel erosion *in vitro*: a comparison of amine, sodium and stannous fluoride. Caries Res.

[18] Rugg-Gunn A, Banoczy J (2013). Fluoride toothpastes and fluoride mouthrinses for home use. Acta medica academica.

[19] Petzold M (2001). The influence of different fluoride compounds and treatment conditions on dental enamel: a descriptive *in vitro* study of the CaF(2) precipitation and microstructure. Caries Res.

[20] Schlueter N, Klimek J, Ganss C (2009). Efficacy of an experimental tin-F-containing solution in erosive tissue loss in enamel and dentine *in situ*. Caries Res.

[21] Ganss C, Neutard L, von Hinckeldey J, Klimek J, Schlueter N (2010). Efficacy of a tin/fluoride rinse: a randomized *in situ* trial on erosion. Journal of dental research.

[22] Ramos-Oliveira TM (2017). AmF/NaF/SnCl2 solution reduces *in situ* enamel erosion - profilometry and cross-sectional nanoindentation analysis. Brazilian oral research.

[23] Wegehaupt FJ, Taubock TT, Sener B, Attin T (2012). Retention of KOH-soluble fluoride formed after application of a SnCl(2)/AmF/NaF containing mouth rinse under erosive conditions. Acta odontologica Scandinavica.

[24] Faller RV, Eversole SL (2014). Protective effects of SnF2 - Part III. Mechanism of barrier layer attachment. International dental journal.

[25] Ellingsen JE (1986). Scanning electron microscope and electron microprobe study of reactions of stannous fluoride and stannous chloride with dental enamel. Scandinavian journal of dental research.

[26] Schlueter N, Klimek J, Ganss C (2009). *In vitro* efficacy of experimental tin- and fluoride-containing mouth rinses as anti-erosive agents in enamel. Journal of dentistry.

[27] Huysmans MC, Young A, Ganss C (2014). The role of fluoride in erosion therapy. Monographs in oral science.

[28] Algarni AA (2015). The impact of stannous, fluoride ions and its combination on enamel pellicle proteome and dental erosion prevention. PloS one.

[29] Hertel S (2017). Effect of Tannic Acid on the Protective Properties of the *in situ* Formed Pellicle. Caries Res.

[30] Weber MT, Hannig M, Pötschke S, Höhne F, Hannig C (2015). Application of Plant Extracts for the Prevention of Dental Erosion: An *in situ*/*in vitro* Study. Caries Res.

[31] Kensche A (2016). Influence of Calcium Phosphate and Apatite Containing Products on Enamel Erosion. The Scientific World Journal.

[32] Hannig C, Becker K, Yankeu-Ngalene VE, Attin T (2008). Applicability of common methods for short time erosion analysis *in vitro*. Oral health & preventive dentistry.

[33] Kensche A, Basche S, Bowen WH, Hannig M, Hannig C (2013). Fluorescence microscopic visualization of non cellular components during initial bioadhesion *in situ*. Arch Oral Biol.

[34] Kensche A (2017). Efficacy of a mouthrinse based on hydroxyapatite to reduce initial bacterial colonisation *in situ*. Arch Oral Biol.

[35] Sicca C, Bobbio E, Quartuccio N, Nicolo G, Cistaro A (2016). Prevention of dental caries: A review of effective treatments. Journal of clinical and experimental dentistry.

[36] Marinho VC, Chong LY, Worthington HV, Walsh T (2016). Fluoride mouthrinses for preventing dental caries in children and adolescents. The Cochrane database of systematic reviews.

[37] Eversole SL, Saunders-Burkhardt K, Faller RV (2015). Erosion Prevention Potential of an Over-the-Counter Stabilized SnF2 Dentifrice Compared to 5000 ppm F Prescription-Strength Products. The Journal of clinical dentistry.

[38] Schlueter N, Klimek J, Ganss C (2011). Efficacy of tin-containing solutions on erosive mineral loss in enamel and dentine *in situ*. Clinical oral investigations.

[39] Schlueter N (2009). Tin-containing fluoride solutions as anti-erosive agents in enamel: an *in vitro* tin-uptake, tissue-loss, and scanning electron micrograph study. Eur J Oral Sci.

[40] Naumova EA (2016). Dynamics of Fluoride Bioavailability in the Biofilms of Different Oral Surfaces after Amine Fluoride and Sodium Fluoride Application. Scientific reports.

[41] Hannig C, Hannig M (2009). The oral cavity-a key system to understand substratum-dependent bioadhesion on solid surfaces in man. Clinical oral investigations.

[42] Reddy A, Norris DF, Momeni SS, Waldo B, Ruby JD (2016). The pH of beverages in the United States. Journal of the American Dental Association.

[43] Van Loveren, C. Antimicrobial activity of fluoride and its *in vivo* importance: identification of research questions. *Caries Re*s **35** Suppl 1, 65–70, doi:49114 (2001). 10.1159/000049114 11359062

[44] van der Mei HC, Engels E, de Vries J, Busscher HJ (2008). Effects of amine fluoride on biofilm growth and salivary pellicles. Caries Res.

